# Individuals Age Determination from Human Dental Pulp Through DNA Analysis by PCR

**DOI:** 10.1055/s-0041-1723084

**Published:** 2021-02-15

**Authors:** M.L. Avinash Tejasvi, Anulekha Avinash CK, E. Rajendra Reddy, Pavan Kulkarni, Harsha Bhayya, Manohar S. Kugaji

**Affiliations:** 1 Department of Oral Medicine and Radiology, Kamineni Institute of Dental Sciences, Narketpally, Telangana, India; 2 Department of Prosthodontics, Kamineni Institute of Dental Sciences, Narketpally, Telangana, India; 3 Department of Pedodontics, Kamineni Institute Dental Sciences, Narketpally, Telangana, India; 4 Department of Oral Pathology, Kamineni Institute of Dental Sciences, Narketpally, Telangana, India; 5 Department of Genetics, Central Research Laboratory, Maratha Mandal's NGH Institute of Dental Sciences and Research Centre, Belagavi, Karnataka, India

**Keywords:** age, PCR, pulp, dentin

## Abstract

**Objectives** Age estimation in forensic odontology is having a great importance in recent times because of the request by court or other government authorities so that immigrants whose real age is unknown should not suffer unfair disadvantages because of their supposed age, and so that all legal procedures to which an individual's age is relevant can be properly followed.

**Purpose** The present study was planned to be conducted on pulp tissue and dental hard tissues derived from individuals for DNA isolation and age determination .

**Materials and Methods** The present study was an experimental single-blinded study consisting of 30 extracted teeth categorized into three groups as follows: Group A: 10 to 20 years, Group B: 21 to 30 years, Group C: 31 to 40 years. DNA was isolated from the pulp of each tooth and quantitative polymerase chain reaction (qPCR) for calculating telomere length was performed.

**Results** With increase in age, the length of telomere gets shortened which will be helpful in analyzing the age of the person when morphological and biological remnants are not available except the tooth.

**Conclusion** The present study found that estimation of human age based on the relative TL measured by the real-time quantitative PCR may be a useful method for age prediction, especially when there is no morphologic information in the biological sample. This is the first study to accesses the age of a person by telomere length using dental pulp.

## Introduction

Age is one of the key parameters in establishing a physical characteristic profile of an individual. For biological evidence left in crime scenes such as blood, saliva, hair, etc., the evidence of owner's age can be determined only by DNA extracted from these materials. Previous researchers have found that there are certain DNA regions with specialized characteristic and function called telomere being able to predict age. 1 Estimating tooth age and skeletal age are the two primary methods in age estimation of forensic medicine. But they are often impacted with geographical environment, nutrition, habitation, and ethnologic differences, so the accuracy will be reduced, especially for the adult. With the study of telomere, it is certain that the length of the telomere DNA can reflect the cell division and represent the cell lifespan, and it has some pertinence to the age of the donor, so to measure the length of telomere DNA is a new and valuable method for age estimation in the forensic medicine. 2

Drastic change is seen in the criminal scenario, road traffic/rail/aviation accidents, mass disasters, wars, and the bodies which are found are beyond recognition. When segments of the body or cranial cavity or isolated teeth are found, sex identification becomes the most intriguing, complex, and sometimes controversial challenge. Teeth being the hardest substances in the human body, potentially can survive most of the insults and consequences encountered at death and during decomposition. Tooth pulp remains protected in a hard tissue casing made up of dentin and enamel. So, the present study is planned to be conducted on pulp tissue and dental hard tissues derived from individuals for DNA isolation and age determination. 3

The aim of the present study is to assess the viability of human dental pulp as a source for DNA and to establish a reproducible, simple, and standardized protocol for age determination by polymerase chain reaction (PCR) method.

We adopted O'Callaghan et al's modified method of absolute quantification of telomere length by introducing oligomer standard to generate telomere values. 4

## Materials and Methods

Study sample was calculated by using a formula by using random sampling technique and 30 extracted teeth of known gender and age were taken. The study included tooth samples of known age range from 18 to 40 years. Tooth samples immediately after extraction were taken, in which vital tooth was considered before extraction, but not vital pulp. Permanent tooth samples extracted for orthodontic treatment, impacted teeth or due to periodontal destruction. There were few exclusions from the study such as tooth samples whose age was not known. The study was done exclusively on permanent tooth. Deciduous tooth samples were excluded from the study. As it was a new study, we wanted more quantity of pulp tissues rather than necrotic pulp so teeth with extensive decay involving pulp were excluded. Tooth with wear facets were excluded because most of the time pulp chamber and canal would be calcified in the presence of ware facets restored and RC treated teeth.

The present study was an experimental single-blinded study consisting of 30 extracted teeth categorized into three groups as follows Group A: 10 to 20 years, Group B: 21 to 30 years, Group C: 31 to 40 years. Two investigators were involved in the extraction of pulp and dentine in the study. Initially teeth were washed in 5.2% sodium hypochlorite solution for around 30 seconds, and then the teeth were cleaned and washed again with sterile distilled water for around 30 seconds. The tooth was wiped with cotton and by using hand trimmer with carborundum disk; each tooth was longitudinally sectioned into two halves. The pulp tissue from each tooth was removed by using an endodontic broach no. 21. The derived pulp sample was then put in a sterile Eppendorf tube containing a Tris EDTA buffer (10-mM Tris buffer, 1 mM EDTA, pH 7.5). The tubes were labeled accordingly and stored at normal room temperature.

Samples of pulp were then sent to the Department of Molecular Biology and Immunology to the second observer who did not know the age of the patient, for further procedure. In the next step, DNA extraction procedure (Modified Proteinase-K method) for pulp was done. 2 Briefly, the samples were vortexed and then washed with fresh TE buffer three times. This is followed by the addition of lysis buffer containing 10-mm Tris buffer and 1-mm EDTA and Lysis buffer II containing 50-mm Tris HCl, 50-mm KCL, MgCl _2_ 2.5 mm, Tween 20 0.45%, and nonidet-P 0.45%. Proteinase K (10 mg/mL) was added to degrade the protein contaminants and kept at 60°C in water bath for 2 hours followed by enzyme deactivation by keeping in boiling water bath for 10 minutes. The DNA was collected from the supernatant after centrifugation at 5,000 rpm for 5 minutes.

Quantitative PCR (qPCR) for calculating telomere length was performed as described by O'Callaghan et al. Standard curve is used to calculate absolute telomere length. A standard curve is set up by the dilution of known quantities of a synthesized 84 mer oligonucleotide containing only TTAGGG repeats ( Table 1 ). The amount of telomere sequence in TEL STD is calculated as 1 × 10 ^8^ kb of telomere sequence in TEL STD as determined by using Avogadro's number. A single copy gene (SCG, 36B4) is used as a control for amplification of every sample performed and to determine genome copies per sample. The genome copy per reaction is calculated as 1 × 10 ^9^ diploid genome copies. A serial dilution of TEL STD was performed (1.0 × 10 ^8^ to 1.0 × 10 ^4^ kb telomere sequences). A serial dilution of SCG STD was also performed simultaneously (1 × 10 ^9^ to 1 × 10 ^5^ dilution).

**Table 1:** Oligomers used in the study

	Sequence name	Oligomer sequence (5 to3′)
Standards	Telomere standard	(TTAGGG)14
	36B4 standard	CAGCAAGTGGGAAGGTGTAATCCGTCTCCACAGACAAGGCCAGGACTCGTTTGTACCCGTTGATGATAGAATGGG
PCR primers	teloF	CGGTTTGTTTGGGTTTGGGTTTGGGTTTGGGTTTGGGTT
	teloR	GGCTTGCCTTACCCTTACCCTTACCC TTACCCTTACCCT
	36B4F	CAGCAAGTGGGAAGGTGTAATCC
	36B4R	CCCATTCTATCATCAACGGGTACAA

Source: Adapted from O'Callaghan N et al. 4

Telomere for each sample including TEL STD was amplified by using telomere-specific primers (TeloF and TeloR) by qPCR to get kb/reaction of telomere for each sample. Single copy gene (36B4) for each sample including SCG STD was amplified using SCG-specific primers (36B4F and 36B4R) by qPCR to get diploid genome copy number for each sample.

**Fig. 1:**
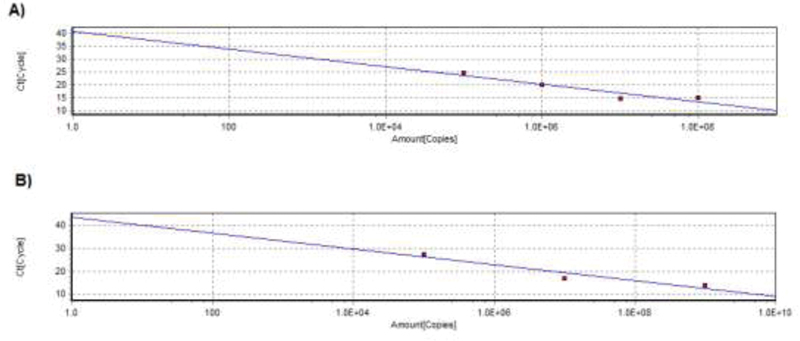
Standard curve used to calculate absolute telomere length. CT (cycle threshold) is the cycle number at which fluorescence signal is generated. ( **A** ) Graph showing standard curve for calculating length of telomere sequence per reaction tube. Slope −3.4 and efficiency is 0.96. ( **B** ) Graph showing standard curve for calculating genome copies using 36B4 copy number. Slope −3.4 and efficiency is 0.95. Standard curves were generated using an Realplex software (Eppendorf, Hamburg, Germany).

CT (cycle threshold) is the cycle number at which fluorescence signal is generated. Telomere standard curve was generated by plotting CT values against amount of telomere sequence in kb per reaction ( Fig. 1A ). SCG standard curve was generated by plotting CT values against 36B4 genome copies ( Fig. 1B ). The standard curves and graphs were generated by using Realplex software (Eppendorf, Hamburg, Germany). the values of telomere kb per reaction and diploid copy number of each sample were exported into excel format. The telomere kb per reaction value was divided by diploid genome copy number to give a total telomeric length in kb per human diploid genome.

## Results

Age determination from tooth pulp was planned and determined through real time (qPCR) in the present study. Group A comprised of 09 samples with an age range of 11 to 20 years, which showed a varied telomere length between 9.57 to 11.05, and a mean telomere length was 9.92 ( Table 2 ). Group B comprised of 17 samples with an age range of 21 to 30 years, which showed a varied telomere length between 8.64 and 10.26, and a mean telomere length was 9.3 ( Table 3 ). Group C comprised of only four samples with an age range of 31 to 40 years, which showed a varied telomere length between 7.32 and 10.25, and a mean telomere length of 9.1275 ( Table 4 ). When the telomere length was compared between the groups, the mean telomere length was found to be decreasing from Group A to Group C. Telomere length was decreased in Group B when compared with Group A and similarly in Group C when compared with Group A and Group B. With the results of present study, we have assessed that, as the age increases the length of telomere gets shortened which will be helpful in analyzing the age of the person when morphological and biological remnants are not available except tooth.

**Table 2:** Group A: 11 to 20 years

Sample no.	Actual age	Telomere length
1	15	9.57
2	15	9.06
3	15	10.04
4	15	9.57
5	18	10.06
6	19	10.09
7	19	10.09
8	20	11.05
9	20	9.88
Mean telomere length		9.92

**Table 3:** Group B: 21–30 years

Sample no.	Actual age	Telomere length
1	21	10.08
2	21	8.64
3	22	10.06
4	23	10.24
5	23	10.12
6	23	10.14
7	23	10.24
8	24	10.26
9	24	9.83
10	24	10.12
11	24	10.26
12	24	9.83
13	24	9.05
14	26	10.14
15	26	10.25
16	29	9.89
17	29	10.25
Mean telomere length		9.3

**Table 4:** Group C: 30–40 y

Sample no	Actual age	Telomere length
1	32	7.32
2	32	9.89
3	35	9.05
4	35	10.25
Mean telomere length		9.1275

## Discussion

There are many methods of estimation of age that are based on anatomical changes such as seen in ribs, hands, and teeth. There are no really good methods for age estimation within the forensic area. A sample of evidence usually carries no morphological information as for instance, a blood stain. In these cases, a method based on molecular observations would be preferred. It has been shown that human telomeres shorten with time. 5 The anatomical position of the dental pulp protects it from various stimuli such as temperature, microbes, or oral fluid. Pulp offers the best source of DNA for reliable genetic analysis in forensic science. DNA is an identical unit of each individual. The smallest amount of DNA can divulge and decipher the biggest mystery. The quality and quantity of pulp tissue will depend on the environmental insult. 6 This is the first study to access the age by telomere length using dental pulp. In the present study, we were able to retrieve pulp tissue in all the groups which were considered. Pulp which was retrieved was sufficient to amplify the DNA in the study. Naik et al 7 was able to retrieve the mean DNA quantity of 26.41 ng/μL. In our study, extracted teeth showed a wide range of DNA quantity. Reason for the same was the varying size of the pulp cavity, which is directly proportional to the age of the participant. Similar to the present study Khan et al, 8 stated in her study that among the total of 20 samples, DNA isolation was done from all the samples of pulp and dentin. Similarly, Battepati and Shodan, 9 in their study achieved 100% positive results in amplifying the DNA quantity extracted from 30 teeth buried in the soil for 2 months.

In our study we showed that the variation in the telomere repeats is considerably observed in dentine pulp tissues. There is no doubt that the telomeres shorten through life, but the degree of this loss varied among different individuals as shown. There will be variations in telomere length in various tissues. What is the cause of the variation seen between humans? Some minor variation might be due to the method itself. However, biological factors such as diseases and lifestyle as well as inherited telomere length and telomerase activity most probably affect the actual telomere length. Several human diseases, in addition to aging in general, are known to be linked to telomeres. In cancer the rate of cell division affects the rate of telomere length. The environment of the cell is also a factor that affects the telomere length external factors such as the level of oxidative stress and the efficiency of the antioxidant defense. Stress and cigarette smoking have been shown to lead to a higher degree of oxidative stress not only these up-regulation of the immune system (i.e., infection) could have an impact on the result. 10

## Conclusion

The present study found that estimation of human age based on the relative TL measured by the real-time quantitative PCR may be a useful method for age prediction, especially when there is no morphologic information in the biological sample. Meanwhile it could only give a rough estimation of age or could be assigned to an age interval. To exactly analyze and determine the age, real-time quantitative PCR assay is simple, rapid, and readily scalable to achieve a high throughput of samples. To exactly analyze the age group of the patient between the age group more number of samples needs to be taken with equal number of samples in each group.
